# Cost-Benefit Analysis of the CT of the Face in the Evaluation of Traumatic Facial Fractures in an Appalachian Tri-state Geriatric Population

**DOI:** 10.7759/cureus.63830

**Published:** 2024-07-04

**Authors:** Gerard V Giangrosso, Ty Bayliss, Armein Rahimpour, Abigail Murphy, Peter Ray, David Denning, Rahman Barry

**Affiliations:** 1 General Surgery, Marshall University Joan C. Edwards School of Medicine, Huntington, USA; 2 Plastic Surgery, King’s Daughters Medical Center, Ashland, USA; 3 Plastic and Reconstructive Surgery, Marshall University Joan C. Edwards School of Medicine, Huntington, USA

**Keywords:** cost-benefit analysis, geriatric trauma, rural appalachia, facial trauma, ct face

## Abstract

Objective: This study aimed to evaluate the cost vs. benefits of the CT face imaging study in the trauma workup of those over the age of 65.

Methods: We performed a retrospective chart review of 169 trauma patients in our trauma database aged 65 years or older who underwent a CT of the head, a CT of the face, or a CT of the head and CT of the face that resulted in findings of a facial fracture from 2017-2022. Injuries and the treatment they received were documented. If a patient underwent both a CT of the face and a CT of the head, then the author first viewed the CT of the head, documented any injury, and then recorded treatment based on the CT of the head. The CT of the face was then viewed, injuries were recorded, and treatment based on the CT of the face was documented. Statistical analysis was then performed using the paired T-test, McNemar test, and number needed to harm analysis.

Results: Of the 169 patients sampled, 159 underwent both CT of the head and the face. There were no patients who underwent a CT of the face exclusively, and only 10 patients underwent a CT of the head exclusively. Of the 159 that had both a CT of the head and the face, the average number of injuries noted on CT of the head + CT of the face vs. CT of the head was 2.42 vs. 1.36, P<.0.0001. The number needed to avoid missing a surgical facial fracture when only a CT of the head was obtained was 14.68.

Conclusion: The risks of missing a surgical facial fracture outweigh the monetary, radiation, and patient-desired necessity benefits of only performing a CT of the head. A CT of the face should be included in the trauma workup for those over the age of 65 when facial fractures are suspected.

## Introduction

The evaluation of a trauma patient is often challenging, even for seasoned clinicians. The establishment of Advanced Trauma Life Support (ATLS) protocols has helped to assess and treat life-threatening injuries efficiently during the primary survey. After initial stabilization, examination typically consists of CT imaging to assess the full extent of the trauma, often a critical part of the secondary survey. This survey routinely involves CT scans of the head, cervical spine, chest, abdomen/pelvis, thoracic spine, and lumbar spine to assess for as many injuries as possible. At our institution, a CT of the face is only obtained when there is obvious severe polytrauma and a patient is unable to voice if they are having facial pain or if the mechanism involved could have caused a facial injury. If a CT of the face is not ordered and an injury is noted or suspected during a tertiary survey or a review of a head CT, then a CT of the face is ordered later.

This is an important note because facial fractures are fairly common in trauma patients. A study by Lalloo et al. showed that the prevalence of facial fractures globally was roughly 23 per 100,000 people in 2017, with 7.5 million new fractures occurring that year [1]. Lalloo et al. also noted that falls were the most common mechanism of injury, and the most common fractures seem to be mandibular fractures, maxillary fractures, zygomatic fractures, and orbital fractures [1, 2]. Facial fractures can also be seen as heralds of more serious injuries, given the high amount of energy that must be required to fracture one of these bones. One study performed by McCarty et al. showed that the presence of an isolated fracture of the facial bones was concomitant with a traumatic brain injury in 21.3%-46% of patients [3]. As such, it is in the best interest of the patient and treating providers to fully assess a patient and determine if they have a facial fracture.

The easiest way to do this is by performing a physical examination with a CT of the face if there is any concern for a fracture. However, many patients seem to question the necessity of getting this imaging study, especially our older, geriatric trauma patients. Most of these people are concerned about three areas regarding the scan: money/cost, radiation, and overall necessity. In the moment, many say they only want surgery in a life-threatening situation, and to them, the face is not an area of vital importance. The primary objective of this study is to evaluate the cost vs. benefit of obtaining a face CT in the trauma workup of patients aged >65 years at a level II trauma center in rural Appalachia.

This work was presented as an oral presentation at the American College of Surgeons (ACS) West Virginia Annual Meeting in May 2023 and as a virtual poster presentation at the American Society of Plastic Surgery General Meeting in October 2023.

## Materials and methods

This retrospective cohort study was started after approval from the institutional review board (IRB) of Marshall University Joan C. Edwards School of Medicine in Huntington, WV (IRB no. 1991492-3). The hospitals approved for this study included two American College of Surgeons-verified level II trauma centers that serve the patients of the tri-state area in western West Virginia, eastern Kentucky, and southern Ohio. All patients who are admitted after a traumatic injury are recorded in our trauma database. A request was made to our trauma coordinators to accumulate all admitted individuals aged 65 years or older who sustained a diagnosed facial fracture using a CT scan of the head, CT scan of the face, or CT scan of both the head and face from 2017-2022. These data were collected and placed into a centralized list utilizing Microsoft Excel (Microsoft® Corp., Redmond, WA). Once this list was generated, the authors performed a chart review to evaluate the injuries sustained by each patient. All patients were de-identified and replaced with arbitrary identification numbers. A total of 174 individuals aged 65 years or older were evaluated in the initial trauma database report. Of these, five patients were inadvertently documented as having a facial fracture when they actually had facial burns. As such, they were removed from the data pool, and their scans were not reviewed.

If a patient only had a CT of the face or CT of the head performed, then the authors would personally review the images of the CT scan and confirm the findings of the injury based on what the radiologist read. The treatment was then recorded. If a patient underwent both a head CT and a face CT, the authors would first review the head CT images and record any fracture noted, regardless of findings by a radiologist. This was done due to our radiologists’ hesitancy to comment on facial fractures based on a dedicated scan used to normally evaluate intracranial abnormalities. The treatment of the fractures based only on the CT head findings was then recorded as if this were the only imaging obtained. The CT of the face was then reviewed, and the findings of the radiologist's interpretation were confirmed. The injuries were documented, and the treatment was then recorded based on the findings of the CT face. Statistical analysis was then performed using the paired T-test, McNemar test, and number needed to harm analysis using SAS (SAS 9.4, SAS Institute Inc., Cary, NC). Statistical significance was defined as a p-value <0.05.

## Results

Of the remaining 169 patients, 10 underwent a CT of the head only. None of these patients required surgery. For eight patients, the recommended treatment was clinical observation. The other two required no treatment at all. A total of 159 patients underwent both head and face CT. The average number of injuries noted on the CT head was 1.357 (std. deviation 1.994), and the average number of injuries noted on the CT face was 2.403 (std. deviation 1.997) (P<0.0001). Based on the CT of the head, 42 patients were treated with observation alone; three were treated with observation in addition to sinus precautions; and one was treated with observation and a soft diet. One patient required a transfer for oculoplastic evaluation, which was not available at our institution. Ninety-eight patients did not need any treatment, either because the injury was not visualized on the CT of the head or because the injury itself did not require any management. Based on the CT of the face, 77 patients were treated with observation, 10 patients were treated with observation and sinus precautions, and seven were treated with observation and a soft diet. The same patient required a transfer for oculoplastic evaluation. The remaining 40 patients did not require any treatment. A summary of the results is organized in Table 1. 

**Table 1:** Treatment of facial fractures based on imaging

	CT Head	CT Face	Sig.	χ^2^ (1, N=159)
None	98	40	P<0.0001	23.543
Observation	42	77	P=0.002	9.714
Observation + sinus precautions	3	10	P=0.010	2.769
Observation + soft diet	1	7	P=0.08	3.125
Transfer	1	1	P=1	0.500
Surgery	14	24	P=0.026	4.97

It should be noted that it is the opinion of the authors that clinical observation is different from no treatment at all. The former implies that both patient and physician actively monitor for lingering, persistent symptoms or cosmetic dissatisfaction with subsequent follow-up, while the latter implies that both patient and physician do not need to actively monitor lasting outcomes from the fracture.

Of critical importance was the number of patients requiring surgery. When reviewing the CT of the head only, 14 of the 159 patients required surgery. When reviewing the CT of the face, 24 patients required surgery. This includes the 14 previously noted on the CT of the head but also includes 10 patients with a surgical fracture missed on the CT of the head alone. This difference between CT of the head and CT of the face was statistically significant: χ2 (1, N = 159) = 4.97, P = 0.026. The number needed to harm in missing a surgical intervention was 14.684, meaning that for every 14 patients that would not have undergone a CT of the face, one surgical fracture would be missed.

The cost of a face CT at our institution is $1968.20, and this is in addition to the amount of radiation that a patient is exposed to. The benefits of getting a CT of the face are not missing a surgical fracture and having an imaging modality for surgical planning if needed. The risk of not getting a CT of the face is mainly missing a surgical fracture, which could lead to facial instability or cosmetic deformities in the future requiring delayed repair. As such, the benefits of identifying a surgical fracture outweigh the risks of missing a surgical fracture, causing a delay in care.

## Discussion

In our study, 169 patients underwent a CT of the head and a CT of the face. We first reviewed the CT head imaging and documented the treatment as if this were our only imaging modality. We then reviewed the CT face imaging and documented the treatment as well. The results of those findings are summarized in Table 1. We decided to separate observation, observation with sinus precautions, and observation with a soft diet since additional layers of precautions fundamentally change how a patient acts with the fracture. This might seem like a small point, but it is a vital one when the difference can be simple observation versus observation and eating strictly soft foods or sneezing with one’s mouth open. With this note in mind, treatment based on the CT of the face changed in 61 out of the 159 patients. Ten patients needed surgery based on the CT of the head alone, while 24 patients needed surgery based on the CT of the face. This included the 10 from the head CT group and 14 new cases that would have been missed if the CT of the head was the only imaging. This difference was statistically significant (p = 0.026). The number needed to harm in regards to missing a surgical fracture was 14.684.

In our population in rural Appalachia, we tend to deal with a particular kind of patient. Many times, the people of this area do not want to be bothered or to bother anyone else. If a procedure or imaging is not necessary, then they do not want to have to deal with it and do not care about the outcomes. This is particularly true when dealing with the elderly in our area. In the trauma bay, it is already a challenge to convince patients to get critical imaging of the head, chest, abdomen, and pelvis, but convincing them to get a CT of the face can be a challenge in and of itself. Their arguments mostly stem from three main points: cost, radiation, and/or necessity. Our main argument with them mostly revolves around missing a surgical fracture and accurate treatment. However, is a CT of the face truly necessary to make a diagnosis and treat accordingly? Can injuries be discovered and treated from a CT of the head alone? Does the extra cost and radiation amount to any significant change in treatment? Prior studies that evaluated the necessity of the CT of the face were all performed in urban populations like Boston, MA, and Taiwan, with no studies being performed in a rural population. This study is the first to evaluate the necessity of the CT of the face in a rural population.

Facial fractures are often a common occurrence. Typically, these injuries occur after falls from standing height or other types of blunt trauma, particularly in older individuals [1]. With nearly 7.5 million new fractures occurring every year, clinicians must be able to identify fractures and treat them as appropriate [1]. The ATLS protocols, individual institution trauma center protocols, and CT imaging have reduced the number of missed injuries at dedicated trauma centers; in one study, Lawson et al. only had three missed maxillofacial injuries in over 26,000 patients [4]. In our study, the average number of injuries seen on the CT of the head alone was 1.994, while the number of facial fractures seen on the CT of the face was 2.403, P<0.0001. This means that nearly one fracture would have been missed if a CT of the head had been ordered only. To a seasoned trauma clinician, this finding is not too surprising. A CT of the head and a CT of the face have different sizes for each individual slice. At our institution, a CT of the head has a slice size of 5 mm, while a CT of the face has a slice size of 2.5 mm. Given the fact that slices are larger on the CT head, it is much more likely to miss the small fractures that could be seen on the CT face. It is also less likely to be able to see the full extent of the injury.

This is essentially the basis of our conversations with patients when the need for a CT of the face arises. In the grand scheme of a polytrauma patient, a fracture of the face may seem trivial. However, missed injuries could mean instability of the face and/or failure of cosmesis. In our study, 10 patients required facial surgery based on their CT head imaging. However, when the CT of the face was reviewed, 24 patients required surgery. This included the original 10, but it also included 14 more who would not have had surgery if only a CT of the head had been obtained. A number-needed-to-harm analysis showed that for every 14.684 people treated with a CT of the head only, one would miss a surgical fracture. In our opinion, this is unacceptable. While not as serious as missing a liver laceration, a splenic laceration, a hollow viscus injury, or an intracerebral hemorrhage, a deformity of the face could have serious social implications for a patient. The face provides subtle changes that help to facilitate social communication between participants, which could be lost if a deformity is not fixed [5]. A patient may have difficulty eating if malocclusion occurs due to a noncorrected mandible fracture. Cosmetically, a lack of facial symmetry could cause increased unwanted social attention. 

Missing a surgical fracture is important to clinicians, but patients often have other concerns when going to the hospital. While often a secondary thought for most physicians, the cost of the procedures and imaging that we perform can significantly impact the lives of patients. A study from the Netherlands in 2019 demonstrated that the average cost per trauma patient was €12,190, or nearly $13,470 in American dollars [6]. In our own system, the average cost of a CT of the face is $1,968.20. This is important because the per capita income of patients in West Virginia is $28,761, and this is below the national average income of $37,638 per the 2020 National Census [7]. Thus, getting a CT of the face can potentially be a huge burden to a patient, when a single admission for a trauma alone can be nearly half of an Appalachian’s entire salary for the year. As such, the cost of the scan is a reasonable argument from a patient’s standpoint.

The amount of radiation that a patient receives is also of concern. Rural Appalachia is dotted with multiple chemical plants that increase a patient’s exposure to carcinogens. Radiation is also a carcinogen, so people in Appalachia are exposed to many factors that increase their risk of cancer. The amount of radiation that a patient is exposed to is reported in various ways: absorbed dose, equivalent dose, or effective dose. At our institution, a CT of the face delivers a whole-body effective dose between 0.7 and 1.6 mSv. This is a relatively small amount of radiation when compared to other CT scans, but in older patients who have more than likely had multiple scans and X-rays, this can be a significant addition, leading to possible malignancy.

Prior studies evaluating the cost vs. benefit of the CT of the face have concluded that the benefits of getting a CT of the face outweigh the costs. Turner et al. found that performing a CT of the face for a trauma evaluation resulted in savings of 22% per patient without sacrificing accuracy [8]. Shubayr et al. concluded that the benefit of obtaining a CT of the head for facial fractures outweighed the radiation risk associated with the scan [9]. Finally, Huang et al. reported that obtaining a CT of the face in trauma patients was significantly beneficial in finding missed fractures in those trauma patients with a traumatic brain injury who obtained a CT of the head [10].

There are a few limitations to this study. First, this study was limited to patients over the age of 65. Further studies should be performed to evaluate the necessity of a CT of the face in pediatrics and for adults younger than 65. Whereas the older patient may have more concerns about the medical necessity of imaging, the younger population may have more concerns about cosmesis and the overall cost. As such, a cost-benefit analysis would be beneficial for these groups and can be elaborated on in future studies. Another limitation of this study involves the inpatient status of our patient population. Since all the patients were taken from a trauma database of admitted patients, we cannot comment on the benefit to those patients who do not get admitted. Another study could be used to evaluate those trauma patients not admitted to the hospital, as the workup of a trauma patient not being admitted to the hospital could influence the conversation between provider and patient in regard to imaging.

## Conclusions

As we have noted, there are valid arguments for and against getting a CT of the face in a trauma workup. The cost and radiation exposure are important factors for the people in rural Appalachia, but from a physician's standpoint, missing a surgical fracture is inexcusable. The potential complications of missing these fractures can result in further problems that could result in even more cost to the patient and/or radiation exposure down the road. Therefore, we recommend that in patients over the age of 65, a CT of the face should be obtained to evaluate facial fractures when there is suspicion of injury.
